# Redesigning Food Handler Training: A Gamified Approach Tested in Italy’s Large-Scale Retail

**DOI:** 10.3390/foods14162803

**Published:** 2025-08-13

**Authors:** Martina Sartoni, Francesca Marconi, Beatrice Torracca, Francesca Pedonese, Roberta Nuvoloni, Alessandra Guidi

**Affiliations:** 1 Department of Veterinary Sciences, University of Pisa, Viale delle Piagge 2, 56124 Pisa, Italy; martina.sartoni@vet.unipi.it (M.S.); francesca.pedonese@unipi.it (F.P.); roberta.nuvoloni@unipi.it (R.N.); alessandra.guidi@unipi.it (A.G.); 2 Interdepartmental Research Center “Nutraceuticals and Food for Health”, University of Pisa, Via del Borghetto 80, 56124 Pisa, Italy

**Keywords:** web app, gamification, training, food handlers, survey, game-based learning, food safety culture, large-scale retail company, food tech, Industry 4.0

## Abstract

Foodborne diseases remain a major global health issue, with over 250 illnesses linked to contaminated food. Effective food safety management relies on well-trained handlers; however, traditional classroom-based, passive learning often lacks engagement and efficacy, limiting awareness and hindering the development of a strong food safety culture. Gamification offers a promising alternative for vocational training, enhancing motivation and engagement through interactive, emotionally engaging learning experiences. This study aims to evaluate the user’s perception of a gamification-based training system (Food Safety Trainer, FST web app) developed and implemented for the training of food handlers in a large-scale retail company in Tuscany, Italy. A total of 249 employees completed a survey after using FST web app for their annual training. Seniority was used as the primary variable to assess differences among respondents. Although some slight variations in opinion emerged, the results indicate that the web app was generally more appreciated than traditional learning. Gamification demonstrated great potential as a tool for enhancing engagement, promoting team building, and supporting the development of a food safety culture. Future studies could extend the evaluation beyond user perception by assessing the system’s effectiveness, comparing outcomes and performance through specific indicators.

## 1. Introduction

Ensuring food safety through effective hygiene practices and a strong food safety culture is a legal obligation for food businesses, fundamental to maintaining safe production environments [1]. Despite these regulatory requirements, foodborne diseases remain a significant global public health issue, often linked to inadequate food handling practices [2]. A crucial strategy to mitigate this risk is to ensure effective training of operators, which plays a key role in ensuring food safety compliance [3]. In the European Union, Regulation (EC) 852/04 [4] mandates food handlers’ training as part of prerequisite programs in food companies. Moreover, Regulation (EU) 2021/382 [5] emphasizes the importance of continuous training as a fundamental pillar in establishing and maintaining a strong food safety culture within organizations. However, several studies highlight challenges in effectively delivering key concepts related to Good Hygiene and Manufacturing Practices (GHPs/GMPs), particularly among newly hired employees [6] or due to barriers such as resistance to change and limited engagement [7]. Additionally, the repetitive nature of mandatory training can act as a deterrent to engagement, reducing attention and retention of key principles, ultimately affecting compliance and effective implementation. This effect is particularly evident in vocational and adult learning [8]. Furthermore, knowledge transfer alone is insufficient to guarantee safe behaviors; regular assessments and motivation-enhancing strategies are needed to bridge training gaps [9,10]. Traditional classroom-based training remains the most employed strategy for the training process at food company level, while interactive approaches such as videos, simulations, and demonstrations are less frequently used [2,11]. However, challenges such as ingrained improper practices, limited resources, and workplace culture hinder long-term effectiveness [12]. Moreover, with regard to the EU regulatory framework, the lack of standardized training requirements has resulted in heterogeneous training programs, and this makes it difficult to assess their impact and effectiveness [13]. To effectively address training shortcomings and harness the potential of innovative approaches, it is crucial to overcome broader systemic barriers, including fragmented governance, limited stakeholders’ collaboration, low awareness, difficulties in behavioral change, and insufficient communication [14].

As a result, there is a growing interest in alternative and innovative methods that enhance engagement, communication and ensure lasting behavioral changes [15]. One promising approach is gamification, which refers to the application of game design elements into non-game contexts to enhance learning and behavioral engagement [16]. Gamification has demonstrated positive outcomes in various fields, including health, mobility, sustainability, and education, by fostering motivation and active participation [17,18]. Typical elements of effective gamification include goal setting, customization, rapid and visual feedback, and social engagement [19]. However, challenges such as operator resistance and potential interference with intrinsic motivation remain [20]. While gamification has been successfully applied and documented in scientific literature within general educational settings, its application in food safety training and related topics has remained underexplored. In recent years, the adoption of gamification-based systems has been increasingly implemented in sectors of healthcare and food services, highlighting their potential for broader applications. Several studies have focused on evaluating the impact of game-based and video game-based systems in reinforcing hygiene and handwashing procedures within the health sector [21,22]. A more recent study conducted by Clark et al. (2020) [23] assessed the perception of a video game designed to promote handwashing habits specifically in foodservice environments, highlighting the crucial role of game mechanics and reward mechanisms in enhancing engagement and behavioral adherence. Targeting young consumers, the study by Crovato et al. (2016) [24] explored the potential of a serious game for food safety, particularly in terms of risk perception and communication among young consumers, and its findings identified a positive correlation between the use of the serious game mechanism and an improved cognitive association. Furthermore, participants demonstrated increased awareness of food-related hazards after engaging with the tool, reinforcing the idea of serious games as an effective medium for risk education. Koch et al. (2022) [25] designed a game-based online intervention targeting approximately 2000 participants aged 20 to 89 in Norway and the United Kingdom. Their study demonstrated that the game-based intervention improved self-reported food safety behaviors and that the effects of the treatment were not limited to younger participants but remained consistent across age groups. More recently, some authors have focused their research on applying gamification strategies to workers in the food supply chain: Rodrigues et al. (2023) [26] developed a case study on the implementation of gamified education to strengthen food safety practices in public markets in Brazil; assessing the level of GHPs/GMPs application before and after training, the study demonstrated the effectiveness of educational game-based strategies in raising awareness on good hygiene and manufacturing practices.

However, to the best of the authors’ knowledge, while some gamification systems have been partially implemented in the context of hygiene practices and food safety, the novelty of this study lies in the systematic and structured implementation of gamification within the specific context of mandatory training programs in large retail environments. Unlike previous studies which often focus on voluntary learning or educational settings, this study addresses a real-world corporate scenario in the food sector, where training is not only mandatory, but a prerequisite in ensuring food safety compliance. Employee engagement in such contexts is typically challenging. This issue is further compounded by distinctive characteristics of the food industry, such as high personnel turnover and workforce variability, particularly within large-scale retail environments. These workforce dynamics necessitate agile, just-in-time training solutions that can efficiently onboard new employees and deliver timely refresher sessions to maintain competency. Additionally, the food industry is in a state of continuous evolution, driven both by frequent updates to regulatory frameworks, and by modifications in company procedures, stemming from advances in food safety science and legislation. Such ongoing developments require systematic revisions of training content and underscore the critical need for recurrent reinforcement to ensure compliance and mitigate risks. Accordingly, gamification strategies must carefully balance engagement and motivational components with the practical demands for scalable, repeatable, and adaptive training interventions. By tailoring gamified elements to this setting and evaluating user perceptions through both quantitative and qualitative data, the study offers new insights into the potential use and applicability of gamification in mandatory workplace training. Specifically, this study aims to evaluate the user’s perception of a gamification-based training system (Food Safety Training, FST, web app) developed and implemented for the training of food handlers in a large-scale retail company in Tuscany, Italy. Based on this premise, this study focused on the implementation of Food Safety Trainer (FST) web app, a gamified training tool designed to improve food handlers’ knowledge and engagement through interactive learning. Developed in collaboration with a large-scale retail company, the FST web app aims to address common training flaws by promoting active learning. The idea here is to evaluate the user perceptions of the gamified approach compared to conventional lecture-based training, contributing to the ongoing discourse on digital learning strategies by highlighting their potential as powerful tools for enhancing food safety education.

## 2. Materials and Methods

### 2.1. Development and Implementation of the Food Safety Trainer Web App

Food Safety Trainer (FST) web app is the result of a collaboration among academic food safety specialists, a large-scale food retail company, and two IT companies (Enigma Tech Srl, Scarperia, Italy, and Creabit Srl, Borgo San Lorenzo, Italy). The FST web app is available at https://foodsafetytrainer.eu, and it was developed with its front-end and back-end components built in parallel. For the visual interface of FST, the front-end, HTML language was used to manage the layout, CSS for the decorations, and JavaScript for the game animations. For the back end, C# was adopted. FST was designed to save and store data of completed gameplay, test duration, and score, for further elaborations. In-cloud data storage was adopted, through RESTful API methodology and compliant with EU General Data Protection Regulation (GDPR) [27].

**Figure 1:**
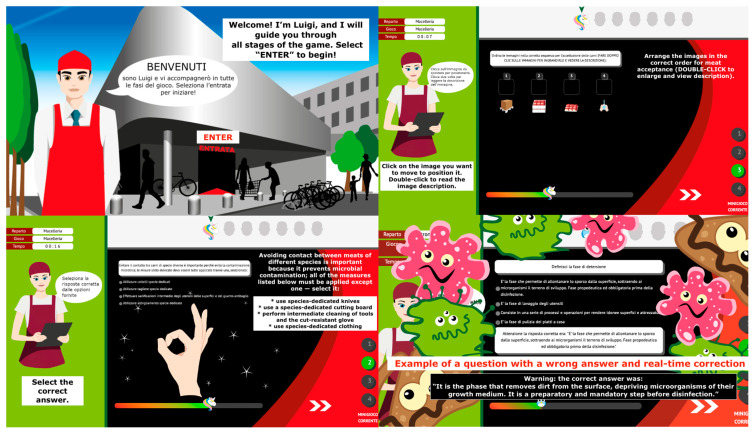
User interface views from the FST web app. The screenshots display key components of the platform, including interactive learning modules, progress monitoring features, and built-in assessment tools.

FST was designed following the principle of ensuring ease of customization for both content and graphics, integrating player avatars to maintain engagement and promote active learning. FST structure included various game mechanics, random question selection, scoring and timing systems, voice reading, and tutorial systems to ensure user-friendliness. Game mechanisms included word input exercises and multiple choice, where users actively insert key terms to reinforce concepts; sequence ordering tasks that encourage logical thinking by having players arrange steps, activities, or ingredients in the correct order, aiding procedural memory; and true/false challenges that promote critical evaluation and quick decision-making. These activities were designed to promote a high level of attention while reducing boredom and passivity, often associated with traditional training methods, which affect knowledge retention. To ensure a coherent alignment between gamification elements and learning objectives, each content area within the FST web app was deliberately addressed through a combination of core game mechanics. This approach was designed to support multiple learning processes—ranging from factual recall to procedural understanding—thereby promoting a deeper and more durable knowledge acquisition. For example, in the contents related to goods receiving, users engaged with both general knowledge questions (via true/false and multiple-choice formats) and context-specific tasks, such as image-sequencing exercises reflecting the correct company procedure. This multimodal strategy allowed for the reinforcement of key concepts like cross-contamination prevention through varied cognitive pathways, ensuring that gameplay not only increased engagement but also strengthened learning outcomes. Time-limited sessions introduced a sense of urgency and focus, increasing cognitive activation and motivation, while the scoring system with scalable cut-off thresholds and the final ranking provided clear goals and feedback, fostering a competitive yet rewarding environment. FST was also designed to provide immediate feedback after each response to support real-time correction and knowledge reinforcement (Figure 1).

**Figure 2:**
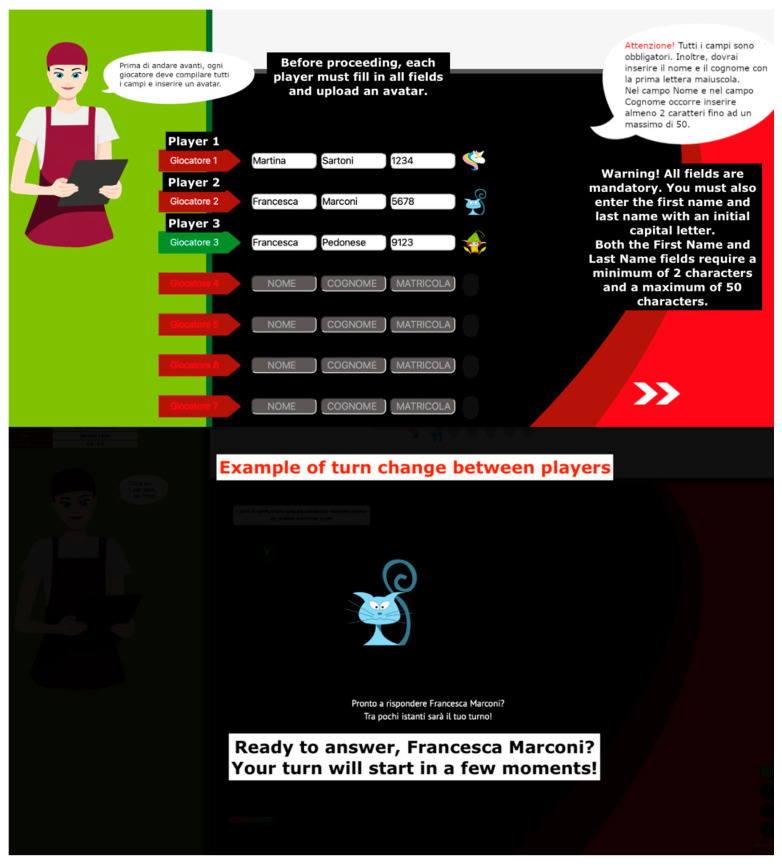
Selection of the number of users and entry of personal details. Example of player turn-taking sequence provided by FST.

The game environment was designed to support both single-player and multiplayer sessions, thereby accommodating diverse training needs—including individual skill development, as well as collaborative learning and team-building activities. Multiplayer sessions were facilitated through a structured participant registration system, followed by a turn-based mechanism in which players were randomly selected to respond to questions (Figure 2). This randomization helped maintain engagement and unpredictability, while also promoting fairness. To ensure consistent participation and prevent disengagement, the system required each player to contribute a minimum number of responses during the session. This dual-layer design—balancing individual accountability with group dynamics—was instrumental in fostering an interactive and inclusive learning atmosphere.

The web app interface is suitable for the use on different devices, such as computers, tablets or smartphones. Within the web app, various training modules were designed and uploaded, encompassing content related to GHPs/GMPs and HACCP. Both general and specific content were developed for individual departments, such as butcheries, fishmongers, bakery or grocery sections, addressing the unique processes, operational practices, and hygiene procedures relevant to each area. These materials considered the particularities and regulatory requirements specific to each department, ensuring targeted and effective training material. Each module included reference to operational instructions and procedures, directly drawn from the company’s food safety management system. The main contents listed by department are presented in Table 1.

FST was initially tested in few stores of the large-scale retailer, reaching a limited sample of employees to conduct some tests. Following these testing phase, the web app underwent minor structural and configuration adjustments and subsequently its use has been extended to all employees, including store managers, department heads, and staff members. The web app was used for the mandatory annual training during the years 2023 and 2024 and that experience is the subject of the investigation in the present study.

### 2.2. Schematic Overview of the Survey Program

**Figure 3:**
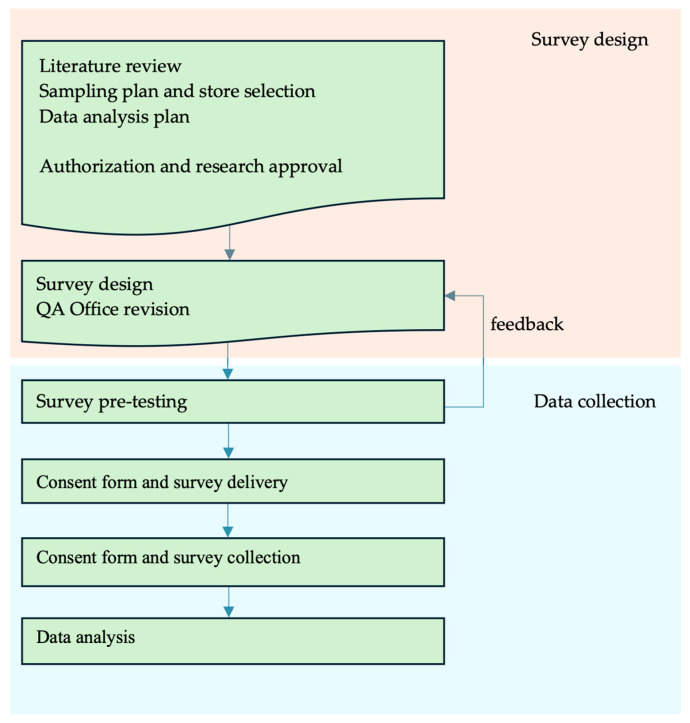
Flow diagram of the survey methodology.

The flow diagram presented in Figure 3 provides a schematic overview of the survey program designed for this study.

The flow diagram highlights the sequential phases of the research process, including the survey planning, the data collection, and analysis. The structured outline of activities is directly aligned with the primary objective of evaluating users’ perceptions of a gamification-based training system (the Food Safety Trainer, FST web app), developed and implemented for training food handlers in a large-scale retail company, and comparing it with conventional lecture-based training.

The administration of a survey was considered the most transparent, and suitable method to investigate the user’s perceptions and simultaneously reach a large number of employees by eliciting their spontaneous, voluntary and anonymous answers. A large-scale retail company was selected due to its significant market share, organizational complexity, and active engagement in food quality and safety research. The company’s interest in partnering to develop and implement the FST web app—prompted by concerns over the effectiveness of its traditional training model—made it a particularly relevant case. Its structure, comprising numerous retail stores and diversified internal processes, was deemed representative of the training needs of similar organizations, also offering a potential model for such type of companies across the region and beyond. Although the research was conducted in a structured company, the outcomes can provide valid insights also for simpler food organizations. Additionally, the involvement of a structured company enabled access to a large and diverse population of food handlers, varying in age and tenure, all subject to mandatory food safety training.

Authorization to conduct the research within the premises and operations of the company was formally granted by the management of the retail organization, enabling full access to relevant data and personnel.

### 2.3. Survey Design and Administration

The survey contents were designed following the model proposed by Clark et al. (2020) [23] and re-elaborated by the research team together with the company’s Quality Assurance (QA) Office. The investigation was approved by the company’s management, ensuring anonymity for all collected data to solicit spontaneous answers and reduce the potential internal or external conditioning. Together with the survey administration, respondents were provided with a concise information sheet outlining the study’s objectives and including a consent form for data usage and processing. The survey protocol was first pre-tested with two members of the research team and subsequently with one member of the (QA) office of the large retailer, to refine the clarity of the questions.

The survey consisted of an introduction and 4 sections. The introduction contained general questions, requiring respondents to provide basic demographic information, such as age group, gender, department and working area, and length of service (<4 years, 4–10 years and >10 years). Sections 1–4 represented the core of the survey aimed at investigating, respectively, the perception of the importance of workplace training (S1), the preference for gamification over traditional training (S2), the perceived enjoyment (S3), and the perceived usefulness (S4). These sections were adapted from those proposed by Clark et al. (2020) [23], as the structure was considered suitable for evaluating both the overall user’s perception on training and the specific opinion on gamification vs. traditional training. Each section contained several statements, for which respondents were asked to provide a rating using a 5-point Likert scale (1 = full disagreement, 5 = full agreement). At the end of the survey, an open field was also provided for any notes and open observations that participants wished to leave regarding the study and the training system.

The survey was administered in paper format and made available to 500 employees, accompanied by an informed consent form outlining privacy and data management information. The choice of paper-based administration was necessary because only department managers had company email accounts. The staff did not have work email addresses, and using personal channels would have raised privacy concerns. Participation in the survey was encouraged by the company itself, but was ultimately left voluntary for employees. The questionnaires were distributed in May 2024, allowing approximately 2 weeks for completion. At the end of this period, the paper-based questionnaires were collected in a single session by a member of the research team from the various retail stores included in the survey.

### 2.4. Statistical Analysis

For each statement and section, the Likert scale scores as given by employees were analyzed by calculating the mode, median, and mean values. Since the data were not normally distributed, non-parametric statistical tests were performed to assess statistical differences among different sections, questions and employees’ groups, using the Kruskal–Wallis test, followed by Wilcoxon test with Bonferroni correction for pairwise comparison. All results were considered statistically significant if associated with a corrected *p*-value < 0.05. Also, the internal reliability of the survey themes was tested determining Cronbach’s α coefficient, using Stata V17.0 (StataCorp LLC, College Station, TX, USA), in accordance with Louangrath (2018) [28]. Cronbach’s α values range between 0 and 1, with values close to 1 indicating strongly associated ratings [29].

## 3. Results

Out of 500 questionnaires distributed, 249 were returned, within the designated timeframe, representing nearly half of the targeted sample. Of the data collected, those obtained from the store managers and department heads accounted for about 5 % of participants—that is, approximately 1 in 20 staff members. Table 2 presents the overall results of the survey, highlighting a strong appreciation for corporate training and for the use of gamification, with mean scores showing a preference for interactive training approaches over traditional methods.

Regarding the first section, Importance of Workplace Training, our findings indicate that training, regardless of the methodology, is perceived as an essential aspect of employees’ work. This is evidenced by statement S1.1, “I believe that receiving training and instruction relevant to my job is important”, which received a very high mean score (M = 4.71). However, lower scores for S1.2 “I believe that the training and instruction I receive are sufficiently specific and aligned with my duties” (M = 4.16) and S1.3 “I believe that the company dedicates adequate time to my training” (M = 4.18) indicate potential areas for improvement in terms of training specificity, customization and duration.

During the analysis, demographic variables such as role, department, and age group were initially considered. However, the distribution within roles and departments proved to be too heterogeneous, making it difficult to identify consistent or meaningful patterns across groups. Regarding age, due to privacy considerations, data were collected identifying only three broad age ranges that resulted very dishomogeneous in numerosity which limited the depth of analysis. Given these constraints, length of service emerged as the most reliable and insightful indicator of cumulative training exposure, compared to the other variables. This is consistent with the study’s aim to compare perceptions of two educational systems. Table 3 presents the results by length of service: this variable significantly influenced perceptions (*p* < 0.00001) with employees having more than 10 years of experience reporting higher average scores across all sections.

In Section S1, which measured the perceived importance of workplace training, the most experienced group reported a significantly higher (*p* ≤ 0.0108) average score (M = 4.42) compared to those with less than 4 years of experience (M = 4.26) and those with 4–10 years of experience (M = 4.16). Similarly, for S3 on perceived enjoyment, employees with over 10 years of service reported an average score of 4.08, noticeably higher (*p* ≤ 0.0027) than the less experienced groups (M ≤ 3.88). The preference for gamification-based training is evident, particularly in S2, preference for gamification over traditional training, where employees with over 10 years of experience achieved an average score of 3.97, compared to M = 3.67 (*p* = 0.0037) for employees with less than 4 years of experience and M = 3.84 for those with 4–10 years. Additionally, S3 revealed a preference for the interactive elements of gamification, with the highest score recorded in statement 3.3 “I find that the colorful graphics, avatars, sound effects, teams, and animations of the game make the training experience more enjoyable and fun” (M = 4.05). However, employees with less than 4 years of experience exhibited significantly lower, albeit still positive, scores in this section (M = 3.65), suggesting that they may struggle to connect the innovative training content with their daily work practices. This result may be due to limited exposure to traditional training models and fewer opportunities for longitudinal comparison. Moreover, newer staff may prefer less interactive and more individually oriented formats, possibly due to reduced self-confidence and a heightened concern about making mistakes in group settings. A tendency to favor broader, less targeted training approaches may have further limited their engagement with content requiring contextual application. Regarding S4 (usefulness), the overall high scores indicated a strong appreciation for gamified training, with statement 4.4 “I believe that the interactive, computerized, and innovative approach has been useful in improving the learning of food safety concepts” receiving M = 4.03. Notably, statement 4.6 “I believe that a training program delivered through team-based gamification would improve teamwork among colleagues and between colleagues and supervisors” obtained a mean score of 4.04; this highlights the perceived benefits of gamification in promoting team building and strengthening collaboration both among peers and between employees and supervisors.

Finally, the open-ended feedback voluntarily provided by some participants in the section at the end of the questionnaire was analyzed thematically and is presented in Table 4. Open feedback on the FST use was mixed. Although the majority of responses were positive, a small number of negative comments, particularly concerning the ease of use and user-friendliness of the interface, were carefully reviewed by the research team together with the company’s QA Office and IT company. The issues reported by employees in the open feedback are consistent with the challenges highlighted in the literature [30,31]. In response, enhancements were made by reviewing content clarity, improving the font size, optimizing the timing between questions, and by developing a tutorial to guide users through the game session. Furthermore, an annual review of the FST product has been established to ensure and support continuous improvement based on QA continuous overview of user feedback and usability.

## 4. Discussion

The objective was to conduct a preliminary exploratory study on the application of a web app related to mandatory training for food handlers. The use of a questionnaire was identified as the most immediate and transparent approach to initiate investigation of the topic, considering that previous studies have already developed surveys to assess perceptions regarding food hygiene training [32,33,34,35] or food safety culture [36,37,38]. The administration of 500 questionnaires resulted in the collection of 249 responses, but potential non-response biases were difficult to identify. On one hand it is possible that only the more motivated or skilled employees, who were more willing to complete the survey, participated; however, this cannot be verified. Additionally, the paper-based administration method may have partially failed to reach some staff members. Therefore, these biases remain challenging to detect. Nevertheless, the fact that only half of the employees participated in a survey on food safety and training topics might arise a reflection about varying degrees of motivation or awareness, highlighting opportunities to further strengthen commitment and knowledge on food safety culture within the organization. As highlighted by Okpala and Korzeniowska (2023) [39], the successful implementation of food safety management systems is often hindered by internal barriers, including limited staff training, lack of awareness, and insufficient organizational commitment. Addressing these factors through targeted engagement and education strategies is essential to promote a shared responsibility for food safety and support the development of an effective food safety culture.

The study’s findings highlight the potentiality of gamification as an effective training tool, particularly in the food sector, where continuous learning is essential for maintaining high safety standards. Results align with existing literature emphasizing the effectiveness of gamification in training. Rodríguez-García et al. (2024) [40] demonstrated that gamification enhances engagement and motivation, leading to higher satisfaction compared to traditional methods. This is consistent with corporate training studies [41] who found that gamification improves compliance program effectiveness, and highlighted its role in enhancing skill acquisition and interest [42]. In the educational sector, other authors [43,44,45,46] emphasized the value of game-based learning in improving knowledge retention and skills.

The positive evaluation of training importance observed in the current study (Statement 1.1, M = 4.71) also suggests a solid food safety culture among employees, contradicting previous findings in the literature [47,48] who reported an optimism bias that led employees to underestimate the impact of training on food safety. However, the scores regarding preference of gamification over traditional training (Statements S2.2 e 2.2, M = 3.89 e M = 3.84) and the open comments left by employees indicate that, as Yertas (2024) [49] suggests, training programs should be highly tailored to job-specific needs to maximize productivity and operational efficiency.

The influence of experience on training perception is another significant finding. More experienced employees reported higher scores in usefulness, motivation, and learning effectiveness (Statements 2.1 M = 3.93, 2.2 M = 3.91, 3.1 M = 3.98, 3.4 M = 4.05), aligning with a previous study [48] that argues that experienced workers recognize the strategic value of new educational methods and integrate them more effectively with existing skills. Additionally, Landers et al. (2019) [50] highlight that gamification can be particularly engaging for senior employees, allowing them to reinforce their knowledge while benefiting from innovative methodologies. Conversely, the lower scores among less experienced workers, particularly in enjoyment (Section 3, M = 3.65), suggest that they may struggle to connect gamified content with daily tasks. In the literature, Insfran-Rivarola et al. (2020) [2] claim that senior employees benefit the most from changes in training delivery methods after years of exposure to repetitive formats, making them more receptive to innovative approaches. Educational research offers several theoretical insights that might help explain why employees with longer tenure may be more receptive and open to gamification. Zhang et al. (2025) [51] highlight that adult learners tend to prefer interactive and autonomous learning formats, particularly when past experiences have been shaped by passive, top-down training methods. This preference aligns with the affordances of gamification. Similarly, Abedini et al. (2024) [52] emphasize that self-efficacy, digital familiarity, and perceived relevance are key personal and behavioral drivers in adult learning, which may develop more strongly over time in experienced employees. Building on this, Pan et al. (2024) [53], through Expectation Confirmation Theory, show that sustained engagement with learning technologies is driven by perceived usefulness and satisfaction—two aspects that gamified environments often fulfill, especially when contrasted with traditional approaches.

Additionally, the high scores in perceived usefulness (Statement 4.4, M = 4.03) and teamwork benefits (Statement 4.6, M = 4.04) confirm previous findings who emphasize how gamification fosters collaboration and a team-oriented work culture [54], as remarked also in some open comments left by some employees. This aspect is particularly relevant in the food industry, where teamwork is essential for maintaining high safety standards. In fact, as pointed out by Okpala and Korzeniowska (2023) [39], the effectiveness of food quality and hygiene systems relies heavily on coordinated, collective practices, where employee training, empowerment, and teamwork serve as fundamental pillars to ensure consistent compliance and process integrity throughout the production chain. Within such frameworks, quality and safety standards are not only technical requirements but shared responsibilities that demand collaborative engagement across all levels of the organization.

However, some criticisms emerged, particularly concerning the lack of verbal interaction and technical limitations of the program. These issues highlight the importance of optimizing gamification tools to ensure accessibility and functional robustness across different devices. Furthermore, as An (2020) [55] suggests, the success of gamified training depends on careful program design, tailored to users’ specific needs and work contexts.

Nonetheless, the study findings indicate that the web app design was particularly effective in fostering motivation and collaborative learning, suggesting gamification’s strong potential for enhancing food safety education. However, future improvements should focus on refining training content to better address the needs of less experienced workers with the adoption of differentiated strategies—such as simplified, guided modules for newcomers and scenario-rich simulations for more experienced staff—and resolving technical limitations. A flexible and adaptive training approach, as suggested by Capatina et al. (2024) [56], could ensure that all employees, regardless of their experience level, benefit fully from gamified training. Moreover, it will be crucial to assess the extent to which game-based learning ensures lasting knowledge retention and improves on-the-job performances. This aligns with current EU regulatory requirements [4,5] and contributes to the development of more effective and inclusive training strategies in the food sector.

Another important consideration is the rapid spread of innovative technologies [57] that have the potential to make training more interactive, immersive, effective, and game-based. A relevant direction for the future development of training tools is the integration of existing digital tools as gamification into immersive technologies, with the goal of enhancing both engagement and learning outcomes. Based on current evidence—rather than speculative technological visions—several features of the FST platform could provide concrete data support and a functional backbone for the expansion towards Augmented Reality (AR)/Virtual Reality (VR)-based training environments also supported by Artificial Intelligence (AI). These include FST modular and hierarchical structure, user-customized learning paths, level-based progression, and flexible session formats (single or multiple). The model in which ‘error becomes a learning opportunity’, characteristic of FST, can also be further developed within an AR/VR/AI scenario. Additional transferable components include access control and privacy management systems, training history tracking, transparent monitoring of completed activities, and the automated generation of training certificates upon completion. These elements not only enhance the learning experience but also fulfill key requirements for traceability and transparency—essential in food safety training programs.

Indeed, VR is one of the most promising technologies. VR can create simulated environments that accurately replicate real working conditions, such as a food processing plant or a production line. Within these virtual environments, operators can practice handling critical situations, such as non-compliance controls or hygiene-related emergencies, without disrupting actual operations. This approach not only enhances practical skills but also reduces the costs and risks associated with on-site training. AR also offers significant opportunities to improve training. The overlay of digital information onto physical environments enables operators to access real-time visual instructions during simulations or practical exercises. For example, AR can be used to display interactive checklists, highlight critical control points (CCPs), or provide real-time guidance on standard operating procedures. This type of contextualized learning enhances the effectiveness and autonomy of trainees, allowing them to consolidate their knowledge in a practical and intuitive way [58,59]. AI represents another key tool for personalizing training pathways. Advanced AI algorithms can analyze participants’ performance, identify areas for improvement, and suggest targeted learning content [60]. Additionally, AI-driven virtual tutors can provide continuous and personalized feedback, making the learning process more dynamic and engaging. AI can also facilitate the development of adaptive simulations, where scenarios evolve based on participants’ decisions, offering a highly immersive and relevant training experience [61]. This adaptive approach could enhance the effectiveness of training by focusing on areas where learners demonstrate the greatest need for improvement.

E-learning platforms, when enhanced with gamification elements, continue to be a versatile solution for training. These platforms allow participants to access content anytime and from anywhere, making training more accessible, particularly for small operators or companies in remote areas. However, special attention should also be given to digital accessibility, ensuring that gamified training tools are usable by workers with varying levels of digital literacy, language proficiency, or physical abilities. This is essential for fostering equity and inclusion across a diverse workforce in the food sector [54]. Another promising pathway is serious games, specifically designed for educational purposes. These games simulate complex scenarios and real-life situations, such as managing a food safety crisis or executing a product recall for non-compliance. Through an immersive approach, participants can practice in safe environments, developing practical skills and improving their decision-making abilities. This type of training is particularly effective for addressing high-risk situations, where errors could have significant consequences.

Taken together, these innovative digital technologies hold significant potential to support training and upskilling in the food industry, offering tools for improved learning, knowledge transfer, and retention. However, the integration of such solutions remains a substantial challenge. As highlighted by recent studies [62,63], the food sector still faces several techno-managerial barriers, including heterogenous digital infrastructure, the necessity of having skilled and regularly updated personnel, and difficulties in identifying digital products that are both tailored and adaptable to specific company needs. Financial constraints further limit adoption, alongside persistent concerns over secure data management. These factors collectively highlight the need for a strategic and context-specific approach to embrace the opportunity of digital innovation in food industry training and operations.

## 5. Conclusions and Future Research Directions

In conclusion, gamification and innovative technologies represent a remarkable opportunity to modernize training in the food sector. The combination of these advanced technologies with gamification opens new perspectives for the training of food industry professionals, fostering a more participatory, dynamic, and continuous learning process. In addition, digital platforms generate valuable data that can be used to monitor learning trends, identify common knowledge gaps, and tailor training content in real time, supporting continuous improvement. When integrated into broader food safety management systems, gamified training aligns learning objectives with operational procedures and compliance requirements, transforming training into a strategic tool for proactive risk management and ongoing quality enhancement. Innovation in training not only enhances workforce awareness and competencies but also contributes to a more efficient resource management, reducing the environmental impact associated with traditional in-person training and travel. The integration of gamification into digital platforms enables continuous and accessible learning, eliminating the need for physical resources such as paper and printed materials, thereby reducing waste and promoting sustainability. However, some challenges must be addressed, including the economic accessibility of advanced technologies and the need to ensure full compliance with existing regulations [62]. For these reasons, investing in innovative training methodologies means not only enhancing workforce skills, but also contributing to the development of a safer, more sustainable, and more resilient food supply chain.

Although the results are promising, this study presents certain limitations, particularly related to the small sample size and a specific context, which may limit the generalizability of the findings. However, the integration of data collected from questionnaires with qualitative feedback provided by users offers valuable insights on the “input–attitude” dimension that opens for future analysis. This could lead to an in-depth evaluation of the perception-behavior-result relationship and the overall effectiveness of the gamified learning tool. Therefore, given these initial positive outcomes, future research should broaden the scope of the evaluation—moving beyond user perception alone—by incorporating specific performance indicators (e.g., improvement in operational tasks, reduction in internal non compliances), and outcome-based comparisons to thoroughly assess the system’s real operational effectiveness and the short, mid and long term impact on workforce skills. In addition, while this study focuses on a single enterprise case, we highlight the need for broader evaluations. Future research should investigate the suitability of the proposed approach across diverse Food Business Operators (FBOs), particularly small and medium-sized enterprises that often struggle with the burden of food handlers’ training management. Exploring the tool’s implementation in FBOs with different characteristics—such as size, workforce, and operational complexity—would offer valuable insights, as these contextual differences remain central to training design and effectiveness. Furthermore, collaboration among academic institutions, certification bodies, regulatory authorities, technology developers and policymakers will be crucial in reducing barriers to digital innovation, defining best practices and establishing guidelines for the widespread and effective implementation of these innovative tools.

## Figures and Tables

**Table 1:** Training module contents by department.

Department	Major Contents
All Departments	Food safety regulatory framework, Prerequisite Programs general principles, overview of HACCP general principles
Butchery	Goods receiving, microbiological risks of raw meat, prevention of cross-contamination, cold chain management, cleaning and disinfection of knives and work surfaces, stock rotation–chain maintenance during display and storage, freshness indicators, labeling
Fishmongers	Goods receiving, hygiene practices in raw fish handling, risk related to *Anisakis*, cold-chain maintenance during storage and display, freshness indicators, labeling, stock rotation
Bakery Pastry and Bistro	Goods receiving, hygiene during preparation, cooking temperature control, storage of raw ingredients, physical hazards, post-baking handling, allergen risk management, personal hygiene, cross-contamination prevention
Delicatessen	Safe handling of ready-to-eat meals, risk related to *Listeria monocytogenes*, management of hot/cold storage, hygiene of utensils and contact surfaces, stock rotation, allergen risk management
Dry Goods Department	Expiry date control, package integrity checks, shelves cleaning, pest control
Produce	Goods receiving, hygienic handling of fresh produce, risk of soil contamination, proper washing procedures, pest monitoring, stock rotation
Rotisserie and Kitchen	Hazards from inadequate cooling or heating, time–temperature control for food safety, handling of packaged foods, management of damaged products
Information Desk, Checkout Area	Fundamental hygiene principles, appropriate behavior in food areas, management of non-compliance reports, handling of pre-packaged foods, management of damaged products
Quality Support	Practical application of the HACCP procedures, verification of hygiene prerequisites, non-conformance management, internal audits contents

**Table 2:** Results of the survey administered to employees with mode, mean, and median values (uppercase letters indicate significant differences among sections; lowercase letters within the same section indicate significant differences among statements).

**Sections and Statements**		**Mode**	**Mean**	**Median**
Section 1—Importance of Workplace Training ^A^ Cronbach’s α coefficient 0.69		5	4.35	5
1.1	I believe that receiving training and instruction related to my job is important ^a^	5	4.71	5
1.2	I believe that the training and instruction I receive are sufficiently specific and aligned with my duties ^b^	4	4.16	4
1.3	I believe that the company dedicates adequate time to my training ^b^	5	4.18	4
Section 2—Preference for Gamification over Traditional Training ^B^ Cronbach’s α coefficient 0.85		4	3.90	4
2.1	I believe that the new training system “Food Safety Trainer” is beneficial for my learning compared to traditional lectures	4	3.89	4
2.2	If I had to choose, I would continue to prefer training through gamification over the traditional method	4	3.84	4
2.3	I would like to continue using interactive training (online courses, gamification, video courses) both for job-specific instruction and for certification-oriented training	5	3.99	4
Section 3—Enjoyment ^B^ Cronbach’s α coefficient 0.89		4	3.98	4
3.1	I feel more motivated to undergo training through gamification compared to the traditional method	4	3.92	4
3.2	The training experience using FST was enjoyable and engaging	4	4.03	4
3.3	I find that the colorful graphics, avatars, sound effects, team dynamics, and animations make the training experience more enjoyable and fun	5	4.05	4
3.4	Considering that the FST app could also be used as a team-based game, I would like to use it in this way	4	3.91	4
Section 4—Usefulness ^B^ Cronbach’s α coefficient 0.92		4	4.04	4
4.1	The alternation and unpredictability of content motivate me to learn and prevent boredom	4	4.07	4
4.2	I believe that the information acquired during training is useful for improving work practices within departments	5	4.07	4
4.3	Practical gaming and visualizing certain concepts help me understand some topics better than verbal explanations	4	4.04	4
4.4	I believe that the interactive, digital, and innovative approach has been useful in enhancing my understanding of food safety concepts	4	4.03	4
4.5	I believe that an interactive, digital, and innovative approach has been beneficial for the practical application of learned concepts in daily work	4	4.00	4
4.6	I believe that a training program delivered through team-based gamification would improve teamwork among colleagues and between colleagues and supervisors	5	4.04	4

**Table 3:** Results by length of service with mode, mean, and median values (uppercase letters indicate overall significant differences among length of service groups; lowercase letters indicate significant differences for each section among length of service groups).

	<4 years ^B^			4–10 years ^B^			>10 years ^A^		
Section	Mode	Mean	Median	Mode	Mean	Median	Mode	Mean	Median
S1	4	4.26 ^b^	4	5	4.16 ^b^	4	5	4.42 ^a^	5
1.1	5	4.72	5	5	4.40	5	5	4.79	5
1.2	4	4.08	4	4	4.00	4	5	4.21	4
1.3	4	3.97	4	4	4.07	4	5	4.26	5
S2	4	3.67 ^b^	4	4	3.84 ^ab^	4	4	3.97 ^a^	4
2.1	4	3.71	4	4	3.86	4	4	3.93	4
2.2	4	3.55	4	4	3.81	4	5	3.91	4
2.3	4	3.74	4	4	3.86	4	5	4.09	4
S3	4	3.65 ^b^	4	4	3.88 ^b^	4	5	4.08 ^a^	4
3.1	4	3.74	4	4	3.86	4	4	3.98	4
3.2	4	3.69	4	4	3.93	4	5	4.12	4
3.3	4	3.64	4	4	3.95	4	5	4.16	4
3.4	4	3,51	4	4	3.77	4	5	4.05	4
S4	4	3.90 ^b^	4	4	3.94 ^b^	4	4	4.10 ^a^	4
4.1	4	3.82	4	4	4.00	4	4	4.15	4
4.2	4	4.03	4	4	3.81	4	5	4.15	4
4.3	4	4.00	4	4	3.95	4	4	4.08	4
4.4	4	3.90	4	4	3.88	4	4	4.10	4
4.5	4	3.82	4	4	4.00	4	4	4.04	4
4.6	4	3.82	4	4	4.00	4	5	4.10	4

**Table 4:** Employees’ qualitative feedback.

Theme	Description	Participants	Example Quote
Involvement	Positive evaluation: usefulness, enjoyment, engagement, practical approach, team-based interaction	12	“An interactive and enjoyable training approach, more engaging than traditional or individual methods”
Contents	Contents to be improved	7	“I would prefer to have questions more specifically tailored to my department”
Language	Recognized need for simpler language	5	“We need simpler language”
Practical learning	The need for increased practical, field-based training has been identified	3	“Hands-on field training is more beneficial than computer-based training”
FST layout	The platform layout needs improvement	2	“In my opinion, the display on mobile devices, such as tablets and smartphones, should be improved, the font size is currently extremely small”
Verbal interaction	Reported need for increased verbal interaction with the trainer	1	“The lack of verbal interaction may result in unresolved questions or uncertainties”

## Data Availability

The original contributions presented in the study are included in the article, further inquiries can be directed to the corresponding author.
